# Orthohantaviruses in Misiones Province, Northeastern Argentina

**DOI:** 10.3201/eid3007.240183

**Published:** 2024-07

**Authors:** María Victoria Vadell, Eliana Florencia Burgos, Daniela Lamattina, Carla Bellomo, Valeria Martínez, Rocío Coelho, Cecilia Lanzone, Carolina Alicia Labaroni, Laura Tauro, Oscar Daniel Salomón, Isabel Elisa Gómez Villafañe

**Affiliations:** Consejo Nacional de Investigaciones Científicas y Técnicas (CONICET), Buenos Aires, Argentina (M.V. Vadell, E.F. Burgos, D. Lamattina, L. Tauro, O.D. Salomón, I.E.G. Gómez Villafañe);; Instituto Nacional de Medicina Tropical, ANLIS Dr. C.G. Malbrán, Puerto Iguazú, Argentina (M.V. Vadell, E.F. Burgos, D. Lamattina, O.D. Salomón);; Instituto Nacional de Enfermedades Infecciosas, ANLIS Dr. C.G. Malbrán, Buenos Aires (C. Bellomo, V. Martínez, R. Coelho);; Instituto de Biología Subtropical (UNaM–CONICET), Misiones, Argentina (C. Lanzone, C.A. Labaroni, L. Tauro);; Instituto de Ecología, Genética y Evolución de Buenos Aires (CONICET-UBA), Facultad de Ciencias Exactas y Naturales, Universidad de Buenos Aires, Buenos Aires (I.E. Gómez Villafañe)

**Keywords:** viruses, orthohantavirus, *Hantaviridae*, rodents, hantavirus pulmonary syndrome, pathogen-host system, Argentina

## Abstract

Few cases of hantavirus pulmonary syndrome have been reported in northeastern Argentina. However, neighboring areas show a higher incidence, suggesting underreporting. We evaluated the presence of antibodies against orthohantavirus in small rodents throughout Misiones province. Infected *Akodon* affinis *montensis* and *Oligoryzomys nigripes* native rodents were found in protected areas of Misiones.

*Orthohantavirus* is a genus of globally distributed RNA viruses in the family *Hantaviridae*. In the Americas, the viruses are hosted by native rodent species within the Cricetidae family (*1*). Although not all orthohantaviruses cause disease in humans, some genotypes are etiologic agents of hantavirus cardiopulmonary syndrome (HCPS), a serious emerging disease (*1*). Along with Brazil and Chile, Argentina is among the countries in South America with the highest incidence of HCPS (*1*). HCPS cases in the country are distributed in 4 epidemiologic regions; incidence is lowest in the northeast. HCPS cases in that region were first registered in 2003 in the south of Misiones province (*2*). Those cases led to the identification of Lechiguanas virus and Juquitiba virus, 2 *Orthohantavirus andesense*–like genotypes, as etiologic agents, and of *Oligoryzomys nigripes* (black-footed pygmy rice rats) as a reservoir of Juquitiba virus, whereas the host of Lechiguanas virus (presumably *O. flavescens* yellow pygmy rice rats) was not confirmed (*2*). Almost 15 years later, infected montane grass mice (*Akodon montensis*) were detected in north Misiones, implicating this species as a new reservoir for orthohantavirus in Argentina (*3*).

Since 2003, <10 orthohantavirus cases have been diagnosed in Misiones (*4*,*5*). However, the circulation of >1 pathogenic genotype and the presence of 3 known orthohantavirus reservoirs, together with a higher incidence of human cases in neighboring states of Brazil, suggest that HCPS might be underreported in this province (*2*,*3*,*6*). Underreporting is likely a result of the high rates of poverty, rurality, and lack of access to healthcare in Misiones (*7*,*8*), factors that are known to contribute to underreporting of diseases (*9*). To identify areas with a potential for higher risk for HCPS, identifying areas where pathogenic orthohantavirus circulates within the rodent community is crucial. In this study, we sought to estimate the seroprevalence of orthohantavirus (as a proxy for infection) and identify the main hosts in protected areas of Misiones. This research was reviewed and approved by the institutional animal care and use committee of the University of Buenos Aires (Faculty of Natural and Exact Sciences).

## The Study

**Figure 1:**
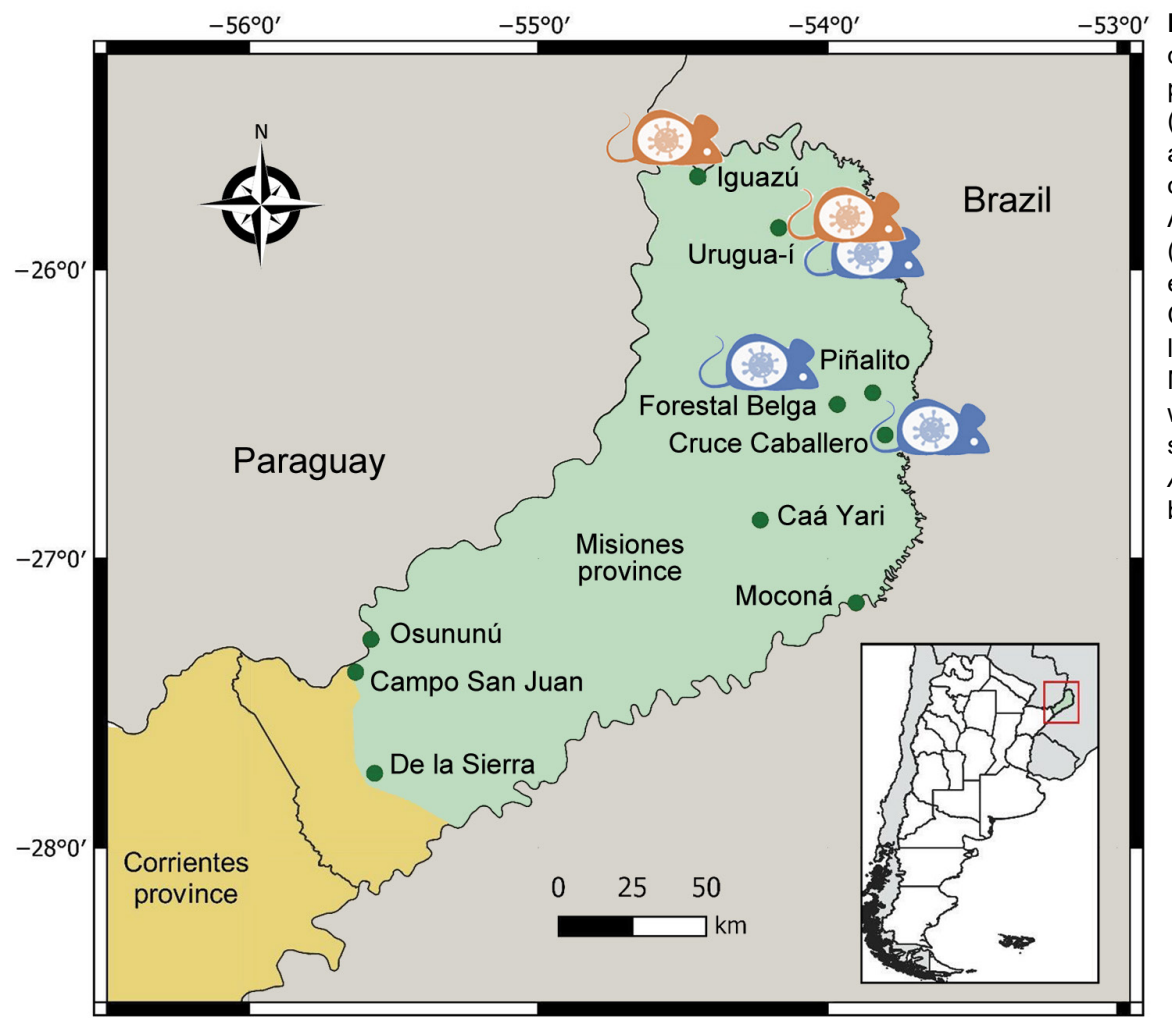
Study areas (green dots) in Misiones Province and part of Corrientes Province (first-level subnational administrative division) in study of orthohantavirus, northeastern Argentina. The Selva Paranaense (Alto Paraná Atlantic Forest) ecoregion is shown in green and Campos y Malezales (savanna-like ecoregion) is shown in yellow. Mouse icons indicate the sites where seropositive *Oligoryzomys* sp. rodents (in orange) and *Akodon* affinis *montensis* mice (in blue) were detected.

We conducted 24 trapping sessions spanning 2–4 consecutive nights in 10 protected areas throughout Misiones Province: Iguazú National Park and Urugua-í Provincial Park in the north; Cruce Caballero, Piñalito, Caá Yarí, and Moconá provincial parks and Forestal Belga protected area in the central part of the province; and Osununú Natural Reserve, Campo San Juan Federal Park, and De las Sierras Provincial Park in the south (Figure 1). We live-trapped rodents during October 2019–February 2023. In each area, we set 60–200 Sherman traps, plus 90 cage traps in some areas, along tracks in the woods. We baited Sherman traps using a mixture of peanut butter, fat, and rolled oats (plus bananas and sardines in most trapping sessions), whereas we baited cage traps with chicken meat and carrots. We identified captured animals up to the last taxonomic level possible according to external morphology. We recorded sex and reproductive conditions of individual rodents. We obtained a blood sample from a small cut on the tip of the tail and later used that sample to analyze the presence of antibodies against orthohantaviruses by using ELISA (*10*). To estimate the diversity of the small rodent community in each study area, we calculated richness, Shannon-Wiener diversity index (−Σp_i_ × ln (p_i_), where p_i_ is the relative proportion of species i in the community), evenness (H/H_max_, where H_max_ = ln[S]), and Simpson diversity index (1 − Σp_i_^2^) using the overall data per trapping area.

A total of 12,424 trap nights yielded 765 rodents of 9 species, resulting in an overall trap success of 6.16%. Species consisted of *A.* affinis *montensis* mice (656), *Oligoryzomys* sp. rodents (28, 1 confirmed as *O. nigripes*), *Sooretamys angouya* (rat-headed rice rat) (23), *Thaptomys nigrita* (blackish grass mouse) (17), *Nectomys squamipes* (scaly-footed water rat) (14), *Euryoryzomys russatus* (big-headed rice rat) (11), *Brucepattersonius iheringi* (Ihering’s akodont) (9), *Oxymycterus quaestor* (quaestor hocicudo) (2), and *Rattus rattus* (black rat) (5) (Table 1).

**Table 1:** Number of captures per species and values of diversity indices in each trapping session and for each area in study of orthohantavirus in Misiones Province, northeastern Argentina*

Study area	Trapping session	Species										Index			
		*Am*	*Tn*	*O*	*Sa*	*Er*	*Bi*	*Oq*	*Ns*	*Rr*		S	H	E	D
Iguazú	2021 Apr	20	0	0	0	0	0	0	0	0		1	0.00	NA	0.00
	2021 Nov	3	0	2	0	0	0	0	0	0		2	0.67	0.97	0.48
	2022 Apr	7	0	0	0	0	0	0	0	0		1	0.00	NA	0.00
	Overall	30	0	2	0	0	0	0	0	0		2	0.23	0.34	0.12
Urugua-í	2021 Jun	24	1	0	0	0	0	0	0	0		2	0.17	0.24	0.08
	2021 Dec	47	1	4	7	0	1	0	0	0		5	0.76	0.47	0.37
	2022 Feb	82	10	2	0	0	0	0	0	0		3	0.44	0.40	0.23
	2022 Jun	62	2	1	0	1	0	0	0	0		4	0.29	0.21	0.12
	Overall	215	14	7	7	1	1	0	0	0		6	0.53	0.29	0.22
Piñalito	2021 Apr	56	0	0	0	0	0	0	0	0		1	0.00	NA	0.00
	2021 Nov†	4	0	2	3	0	0	0	0	0		3	1.06	0.97	0.64
	2022 Apr†	40	1	0	0	0	0	0	0	0		2	0.11	0.17	0.05
	Overall	100	1	2	3	0	0	0	0	0		4	0.27	0.20	0.11
Forestal Belga	2019 Oct†	19	1	2	0	4	1	0	3	0		6	1.20	0.67	0.56
	2021 Oct†	2	0	0	4	0	0	0	0	0		2	0.64	0.92	0.44
	Overall	21	1	2	4	4	1	0	3	0		7	1.37	0.70	0.62
Cruce Caballero	2021 Apr	31	0	0	0	1	1	0	0	0		3	0.27	0.25	0.12
	2021 Nov†	19	1	0	4	1	1	0	0	0		5	0.89	0.55	0.44
	2022 Apr†	54	0	0	0	1	1	0	0	0		3	0.18	0.16	0.07
	Overall	104	1	0	4	3	3	0	0	0		5	0.44	0.27	0.18
Caá Yarí	2021 Aug	6	0	2	0	0	0	0	4	0		3	1.01	0.92	0.61
Moconá	2022 Apr	23	0	3	0	3	4	2	0	0		5	1.11	0.69	0.54
Osununú	2021 Aug	12	0	0	0	0	0	0	0	0		1	0.00	NA	0.00
	2021 Oct	7	0	0	1	0	0	0	0	0		2	0.38	0.54	0.22
	2022 Jan	22	0	0	0	0	0	0	0	0		1	0.00	NA	0.00
	Overall	41	0	0	1	0	0	0	0	0		2	0.11	0.16	0.05
Campo San Juan	2021 Aug	13	0	7	0	0	0	0	5	0		3	1.02	0.93	0.61
	2022 Nov†	40	0	0	0	0	0	0	0	5		2	0.35	0.50	0.20
	2023 Feb†	55	0	1	0	0	0	0	0	0		2	0.09	0.13	0.04
	Overall	108	0	8	0	0	0	0	5	5		4	0.56	0.41	0.26
De las Sierras	2021 Oct	8		2	4		0	0	2	0		4	1.21	0.88	0.66

*Am, *Akodon* affinis *montensis*; *Bi*, *Brucepattersonius iheringi*; D, Simpson index; E, evenness index; *Er*, *Euryoryzomys russatus;* H, Shannon-Wiener index; NA, not applicable; *Ns*, *Nectomys squamipes*; *O*, *Oligoryzomys* sp.; *Oq*, *Oxymycterus quaestor*; *Rr*, *Rattus rattus*; S, richness index; *Sa*, *Sooretamys angouya*; *Tn*, *Thaptomys nigrita*. †Both cage and Sherman traps were used in these trapping sessions (more Sherman traps than cage traps)

We detected antibodies against orthohantavirus in *A.* aff. *montensis* mice with an overall seroprevalence of 0.007; overall seroprevalence in *Oligoryzomys* sp. rodents was 0.083 (Table 2). Seropositive rodents were captured in 4 natural areas, Urugua-í, Cruce Caballero, Iguazú, and Forestal Belga; Urugua-í was the only area in which antibodies were found in both species (Table 2; Figure 1). Because of its relevance to this research, the seropositive *Oligoryzomys* sp. rodent captured in Iguazú National Park was identified at the species level through molecular characterization. We amplified a fragment of the cytochrome b gene (1073 bp) by PCR using primers Mus 14095 and Mus 15398 (*11*). We used BLAST (http://blast.ncbi.nlm.nih.gov) to compare the sequence obtained (GenBank accession no. PP372564) with reference GenBank sequences and identified it as *O. nigripes* (98.71% BLAST identity and 100% coverage).

**Table 2:** Number of trap nights, total captures (xcluding same-session recaptures) and seroprevalence of orthohantavirus in *Akodon* affinis *montensis* and *Oligoryzomys* sp. rodents in each trapping session and overall in each study area in study of orthohantavirus in Misiones Province, northeastern Argentina

Location	Trapping session	Trap nights	Total captures	*A.* aff. *montensis* seroprevalence	*Oligoryzomys* sp. seroprevalence
Iguazú	2021 Apr	300	20	0 (0/20)	0 (0/0)
	2021 Nov	800	5	0 (0/3)	0.5 (1/2)
	2022 Apr	534	7	0 (0/7)	0 (0/0)
Urugua-í	2021 Jun	792	25	0 (0/22)	0 (0/0)
	2021 Dec	800	60	0.021 (1/47)	0.25 (1/4)
	2022 Feb	540	94	0 (0/82)	0 (0/0)
	2022 Jun	800	66	0 (0/57)	0 (0/1)
Piñalito	2021 Apr	400	56	0 (0/54)	0 (0/0)
	2021 Nov*	920	9	0 (0/4)	0 (0/2)
	2022 Apr*	400	41	0 (0/14)	0 (0/0)
Forestal Belga	2019 Oct*	750	30	0 (0/19)	0 (0/2)
	2021 Oct*	720	6	1.0 (1/1)	0 (0/0)
Cruce Caballero	2021 Apr	300	33	0.032 (1/31)	0 (0/0)
	2021 Nov*	920	26	0.071 (1/19)	0 (0/0)
	2022 Apr*	720	56	0 (0/54)	0 (0/0)
Caá Yarí	2021 Aug	390	12	0 (0/5)	0 (0/2)
Moconá	2022 Apr	680	35	0 (0/20)	0 (0/2)
Osununú	2021 Aug	648	12	0 (0/9)	0 (0/0)
	2021 Oct	656	8	0 (0/5)	0 (0/0)
	2022 Jan	776	22	0 (0/21)	0 (0/0)
Campo San Juan	2021 Aug	360	25	0 (0/8)	0 (0/7)
	2022 Nov*	720	45	0 (0/34)	0 (0/0)
	2023 Feb*	720	56	0 (0/47)	0 (0/0)
De las Sierras	2021 Oct	438	16	0 (0/8)	0 (0/2)

*Both cage and Sherman traps were used in this trapping session (more Sherman traps than cage traps).

All seropositive rodents were active males (the sex of 1 seropositive *Oligoryzomys* sp. rodent was not recorded). Overall male-to-female ratio by species was 1.7:1 for *A.* aff. *montensis* and 2.3:1 for *Oligoryzomys* sp.

**Figure 2:**
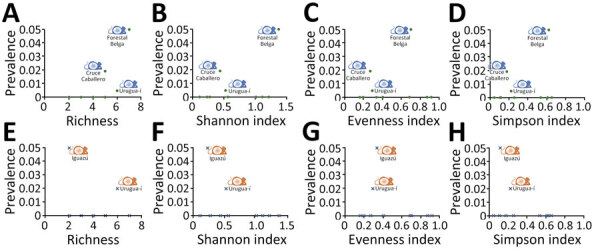
Orthohantavirus seroprevalence in *Akodon* affinis *montensis* mice (green dots) and in *Oligoryzomys* sp. rodents (blue crosses) as a function of richness and Shannon-Wiener, evenness, and Simpson indices in study of orthohantavirus in Misiones Province, northeastern Argentina. Mouse icons indicate presence of seropositive *A.* aff. *montensis* (in blue) and *Oligoryzomys* sp. (in orange) rodents.

Overall seroprevalence in *A.* aff. *montensis* mice was significantly correlated (Spearman test) with richness (ρ = 0.775, p = 0.008) but not with the Shannon-Wiener diversity index (ρ = 0.261, p = 0.466), evenness index (ρ = −0.052, p = 0.886), or Simpson diversity index (ρ = 0.172, p = 0.636) (Figure 2). Overall seroprevalence of *Oligoryzomys* sp. rodents was not significantly correlated with any of the diversity measures (richness, ρ = −0.110, p = 0.762; Shannon-Wiener diversity, ρ = −0.372, p = 0.290; evenness, ρ = −0.164, p = 0.65; Simpson diversity, ρ = −0.277, p = 0.439) (Figure 2).

## Conclusions

Our findings not only expand the known distribution of orthohantavirus in Misiones, Argentina, but also provide evidence of orthohantavirus infection in *O. nigripes* rodents in the north of this province, suggesting the presence of a pathogenic genotype in an area without known human cases. This information is relevant, particularly considering that Iguazú National Park, where 1 seropositive *O. nigripes* rat was captured, is visited by >1 million tourists every year.

Several pathogenic orthohantavirus have been associated with *O. nigripes* rodents and other *Oligoryzomys* spp. rodents in eastern Paraguay, southern Brazil, and northeastern Argentina (*1*,*2*,*12*), suggesting the seropositive animals detected in this study are probably hosts of a pathogenic genotype. However, the possibility of a spillover event from infected *A.* aff. *montensis* mice cannot be ruled out because this species is an orthohantavirus host in north Misiones (*3*). In fact, *Oligoryzomys* spp. rodents and *A.* aff. *montensis* mice were found in sympatry in all but 2 areas, suggesting the high potential for genetic reassortment and host-switching events (*13*), particularly in Urugua-í, where both species were found seropositive. Future studies should aim to identify the orthohantavirus genotypes in these hosts.

Although the male-to-female ratio was close to 2:1 for both species, the fact that all seropositive rodents were reproductively active males supports the role of sex in orthohantavirus transmission (*1*,*3*,*14*). Seroprevalence in *A.* aff. *montensis* mice was positively correlated with richness. However, that evidence is weak because of the low number of sites with seropositive rodents and was not supported by any other diversity measure.

The low overall seroprevalence detected in this study suggests HCPS risk is low in Misiones Province. However, the capacity of cricetid populations to peak unexpectedly under certain conditions (*14*,*15*), in addition to the evidence of orthohantavirus circulation in northern and central Misiones, highlight the potential risk and the need to continue surveillance.

Appendix Additional information about orthohantavirus in Misiones Province, northeastern Argentina
