# Supplementary material for: Orthohantaviruses in Misiones Province, Northeastern Argentina
Source: Emerg Infect Dis. 2024 Jul;30(7):1454–8. doi: 10.3201/eid3007.240183 (PMC11210654; doi:10.3201/eid3007.240183)
Supplement: Appendix — Additional information about orthohantavirus in Misiones Province, northeastern Argentina [file 24-0183-Techapp-s1.pdf]

Article DOI: https://doi.org/10.3201/eid3007.240183

EID cannot ensure accessibility for supplementary materials supplied by authors. Readers who have difficulty accessing supplementary content should contact the authors for assistance.

Orthohantaviruses in Misiones Province, Northeastern Argentina

Appendix

Methodology

We conducted a total of 24 trapping sessions in 10 protected natural areas throughout Misiones province: Iguazú National Park and Urugua-í Provincial Park in the north, Cruce Caballero, Piñalito, Caá Yarí and Moconá provincial parks, and Forestal Belga protected area in the center, and Osununú Natural Reserve, Campo San Juan Federal Park and De las Sierras Provincial Park in the south. Within each natural area, trapping sites were chosen to represent all major habitat types within the area that were suited and accessible for trapping. Rodents were live trapped between October 2019 and February 2023. In each natural area, 60–200 Sherman traps, plus 90 cage traps in certain areas, were set along tracks across the woods. Sherman traps were baited with a mixture of peanut butter, fat, and rolled oats (plus bananas and sardines in most trapping sessions), while cage traps were baited with chicken meat and carrots. A cotton ball was added to each Sherman trap to provide a nesting material to minimize thermal stress. Traps were set active during two-four consecutive nights. Each morning, animals were collected and carried to a field processing station where they were anesthetized and sampled following standardized procedures and biosafety guidelines for working with small mammals (*1,2*). Captured animals were identified by highly-trained researchers up to the last taxonomic level possible according to their external morphology (*3,4*). Individuals were sexed, and their reproductive conditions were recorded (active: scrotal or semi-scrotal testis / opened vaginas; inactive: abdominal testis / closed vaginas). A blood sample was obtained from a small cut on the tip of the tail of each rodent, and placed in an absorbent paper where it dried. These samples

of dried blood were sent to the National Institute of Infectious Diseases (Instituto Nacional de Enfermedades Infecciosas -ANLIS "Dr. C. G. Malbrán") in Buenos Aires for serologic analysis. Rodents were released at the place of capture. Individuals with fresh scars in the tip of their tails were recognized as recaptures of the same trapping session (past few days). In those cases, individuals were released without taking any more samples. All trapping and sampling procedures were reviewed and approved by the Committee on Animal Use of the University of Buenos Aires (Faculty of Natural and Exact Sciences; protocol #125), and were authorized by the ministry of Ecology and Natural Resources of Misiones province (authorizations #23/19, #9/2021, #26/2021, and #31/2022), and by the National Parks Administration of Argentina (authorizations # NEA 506, # NEA 506/1 and # NEA 506/2).

To estimate the diversity of the small rodent community in each study area, we calculated richness (S), Shannon-Wiener (H; $H = - \sum p_i * \ln (p_i)$, where p_i is the relative proportion of species i in the community), Evenness (E; $E = H / H_{max}$, where $H_{max} = \ln (S)$), and Simpson (D; D = 1 - Σpi2) indexes using the overall data per trapping area. Blood was tested for antibody reactive with *Orthohantavirus andesense* (ANDV) recombinant nucleocapsid protein antigen by ELISA as previously described (*5*). Briefly, recombinant ANDV nucleoprotein was used as a specific antigen, the blood samples were diluted 1:100, then incubated with peroxidase-labeled goat anti-*Peromyscus leucopus* IgG secondary antibody (Kirkegaard and Perry Laboratories); ABTS (2.2'-azino-di [3-ethyl-benzthiazoline sulfonate]) was used as the substrate for peroxidase, and absorbance was measured at 405 nm.

References

1. Carroll DS, Tack D, Calisher, CH. Biosafety guidelines for working with small mammals in a field environment. In: Wooley DP, Byers KB, editors. Biological safety: principles and practices. 5th ed. Washington DC: ASM Press; 2017. p. 679–85.

2. Sikes RS, Gannon WL. Guidelines of the American Society of Mammalogists for the use of wild mammals in research. J Mammal. 2011;92:235–53. PubMed https://doi.org/10.1644/10-MAMM-F-355.1

3. Bonvicino, C. R., Oliveira, J. D., & D'Andrea, P. S. (2008). Rodents of Brazil Guidebook, with keys for genera based on external characters [in Portuguese]. Centro Pan-Americano de Febre Aftosa - OPAS/OMS, Rio de Janeiro. Technical Manuals Series #11, 120 pp.

4. Teta P, Jayat JP. Identification keys for murid rodents from Argentina [in Spanish].. Therya. 2021;12:501–26. https://doi.org/10.12933/therya-21-1110

5. Padula P, Martinez VP, Bellomo C, Maidana S, San Juan J, Tagliaferri P, et al. Pathogenic hantaviruses, northeastern Argentina and eastern Paraguay. Emerg Infect Dis. 2007;13:1211–4. PubMed https://doi.org/10.3201/eid1308.061090
